# Genomic Landscape of RTK/RAS Pathway and Tumor Immune Infiltration as Prognostic Indicator of Lung Adenocarcinoma

**DOI:** 10.3389/fonc.2022.924239

**Published:** 2022-07-21

**Authors:** Xiang-Qian Yin, Xue-Hui Yin, Ya-Qin Yu, Lang Xu, Mao Zhang

**Affiliations:** ^1^ Department of Oncology, The People’s Hospital of Huangpi, Wuhan, China; ^2^ Brain Science and Advanced Technology Institute, Wuhan University of Science and Technology, Wuhan, China

**Keywords:** genomics, RTK/RAS pathway, immune infiltration, prognosis, nomogram, lung adenocarcinoma

## Abstract

The RTK/RAS pathway is an oncogenic signaling pathway for which many targeted drugs have been developed; however, survival remains poor. A combination of targeted therapy and immunotherapy has emerged as an option for improving cancer treatment responses. In this study, on the basis of the expression, survival, single nucleotide variation (SNV), copy number variation (CNV), and methylation data of lung adenocarcinoma (LUAD) from The Cancer Genome Atlas database, we comprehensively analyzed the genomic changes in the RTK/RAS pathway and their associations with tumor-infiltrating lymphocytes (TIL) and prognosis in LUAD to provide the genomics landscape of RTK/RAS with TIL and prognosis. We found that two rarely mutated genes, mitogen-activated protein kinase kinase 1 and insulin-like growth factor 1 receptor, were significantly associated with the worse survival of patients with LUAD. Patients with LUAD and co-mutation of KRAS proto-oncogene (*KRAS*) and neurofibromin 1 genes had worse survival, and the underlying mechanism could be insufficient for protein synthesis and intracellular signal deactivation. Methylation of the Rac family small GTPase 1 (*RAC1*) was associated with better survival. The SNVs of the top mutated genes, including epidermal growth factor receptor (12.7%), neurotrophic receptor tyrosine kinase 3 (7.8%), erb-b2 receptor tyrosine kinase 4 (8.5%), and *KRAS* (29.6%), were associated with T cell exhaustion in LUAD. To construct nomograms, we further screened the genes whose genomic changes were closely associated with survival and immune infiltration. The nomograms performed well in predicting disease-specific survival (DSS) with a concordance index of 0.7 (0.589, 0.811) and overall survival with a concordance index of 0.689 (0.603, 0.775) in test set; they also showed good correspondence between actual and ideal nomogram predictions. Tumor stage, *RAC1* methylation, and type 1 regulatory T cells greatly contributed to DSS and OS nomograms. In summary, we provided a comprehensive genomic profile of the RTK/RAS pathway in LUAD and its association with immune cell infiltration and prognosis of LUAD. This profile would serve as a basis for developing better therapeutic strategies, improving patient prognosis, and understanding the mechanisms of immune disturbance from the perspective of oncogenic pathways of LUAD.

## Introduction

Lung cancer is one of the most fatal malignant cancers, accounting for 26% of cancer-related deaths worldwide (1). The most prevalent histological class of lung cancer is non-small-cell lung cancer (NSCLC), with approximately 85% incidence (2), while the most common NSCLC subtype is lung adenocarcinoma (LUAD). The survival rate of patients with LUAD is poor (5-year relative survival: 18%) (1). Many drugs targeting oncogenic pathways, such as the RTK/RAS pathway, have been developed for LUAD; however, the survival of patients with LUAD remains poor (3). Therapeutic failure sometimes occurs because of heterogeneity in the tumor microenvironment (TME). The TME involves interactions between immune and tumor cells and plays a pivotal role in cancer progression and patient prognosis (4, 5). The TME and its immune context may be shaped by oncogenic signaling pathways of tumor cells (6). A combination of oncogenic signaling pathways and the immune system is a promising strategy for cancer therapeutics and prognostic prediction. For instance, the RTK/Ras/PI3K/AKT pathway is a potential biomarker for cancer immunotherapy (7). Mitogen-activated protein/extracellular signal-regulated kinase kinase inhibition and the CD274 molecule/prephenate dehydratase 1 immune pathway elicit a synergistic effect in response to lung cancer therapy (8). The KRAS proto-oncogene (*KRAS*) G12C inhibitor sotorasib combined with immune checkpoint inhibitors produces long-term cure in immune-competent mice (9). These findings suggest that combination therapies have emerged as cancer treatment options. Thus, biomarkers associated with immunity should be screened from the existing oncogenic pathway to help develop better therapeutic strategies and improve patient prognosis.

In this study, we comprehensively analyzed the mutation landscape (single nucleotide variation (SNV), copy number variation (CNV), and methylation) of 38 genes in the RTK/Ras pathway and estimated its association with immunity and four survival types. We found that two rarely mutated genes (*MAPK1* and *IGF1R*) were potential prognostic predictors of LUAD; patients with LUAD had co-mutation of *KRAS* and neurofibromin 1 (*NF1*) genes served worse survival, and the underlying mechanism could be insufficient for protein synthesis and intracellular signal deactivation. These findings providing new targets for precision medicine. We revealed close associations between mutations in the RTK/RAS pathway and TME immune infiltration. These associations could serve as powerful predictors of LUAD survival in nomograms. Our study provided more possibilities for the evaluation of survival probability *via* a mutation-immune combined strategy to help establish better therapeutic strategies, improve patient prognosis, and understand the mechanisms of immunity disturbance from the perspective of oncogenic pathways of LUAD.

## Materials and Methods

### Data Collection and Processing

The 38 key genes of the RTK/RAS pathway were obtained from published studies (10). SNV, CNV, and methylation data were provided by GSCA (11) (http://bioinfo.life.hust.edu.cn/GSCA/#/), the synapse database, and the University of California Santa Cruz Xena (http://xena.ucsc.edu/). In brief, processed SNV data from 565 LUAD tumor samples were collected from the Synapse database (ID: syn7824274). Seven types of SNVs were included in the analysis: missense mutations, nonsense mutations, frameshift insertions, frameshift deletions, splice sites, in-frame deletions, and in-frame insertions. For CNV data, continuous gene-level CNVs were used for correlation analysis. To get the grouped CNV data, the continuous CNV data were further processed using GISTIC2.0 (12) threshold method to obtain categorized CNV data. According to the GISTIC score, the CNV data were divided into five categories: homozygous deletion (score = −2: deep loss), homozygous amplification (score = 2: high-level amplification), diploid (score = 0), heterozygous deletion (score = −1: shallow loss), and heterozygous amplification (score = 1: low-level gain). Based on this, CNV data were further classified into amplification (Amp., homozygous amplification, and heterozygous amplification), deletion (Dele., homozygous deletion, and heterozygous deletion), and wild type (WT). For methylation, Illumina Human Methylation 450k-level 3 methylation data was used for the correlation analysis of each gene to screen the sites that were most negatively correlated with gene expression. Only these sites were included in methylation analysis (**Table S1**). A total of 513, 513, 442, and 278 samples had overall survival (OS), progression-free survival (PFS), disease-specific survival (DSS), and disease-free survival (DFI) data in LUAD. The sample size of each data type was summarized in **Table S2**.

### RTK/RAS Pathway Gene Collection

The following gene sets of the RTK/RAS pathway were collected from a previously published study (10): epidermal growth ;factor receptor (*EGFR*), erb-b2 receptor tyrosine kinase 2 (*ERBB2*), erb-b2 receptor tyrosine kinase 3 (*ERBB3*), erb-b2 receptor tyrosine kinase 4 (*ERBB4*), MET proto-oncogene, receptor tyrosine kinase (*MET*), platelet derived growth factor receptor alpha (*PDGFRA*), fibroblast growth factor receptor 1 (*FGFR1*), fibroblast growth factor receptor 2 (*FGFR2*), fibroblast growth factor receptor 3 (*FGFR3*), fibroblast growth factor receptor 4 (*FGFR4*), KIT proto-oncogene, receptor tyrosine kinase (*KIT*), insulin like growth factor 1 receptor (*IGF1R*), ret proto-oncogene (*RET*), ROS proto-oncogene 1, receptor tyrosine kinase (*ROS1*), ALK receptor tyrosine kinase (*ALK*), fms related receptor tyrosine kinase 3 (*FLT3*), neurotrophic receptor tyrosine kinase 1 (*NTRK1*), neurotrophic receptor tyrosine kinase 2 (*NTRK2*), neurotrophic receptor tyrosine kinase 3 (*NTRK3*), Janus kinase 2 (*JAK2*), Cbl proto-oncogene (*CBL*), ERBB receptor feedback inhibitor 1 (*ERRFI1*), ABL proto-oncogene 1, non-receptor tyrosine kinase (*ABL1*), SOS Ras/Rac guanine nucleotide exchange factor 1 (*SOS1*), *NF1*, RAS p21 protein activator 1 (*RASA1*), protein tyrosine phosphatase non-receptor type 11 (*PTPN11*), *KRAS*, HRas proto-oncogene, GTPase (*HRAS*), NRAS proto-oncogene, GTPase (*NRAS*), Ras like without CAAX 1 (*RIT1*), A-Raf proto-oncogene, serine/threonine kinase (*ARAF*), B-Raf proto-oncogene, serine/threonine kinase (*BRAF*), Raf-1 proto-oncogene, serine/threonine kinase (*RAF1*), Rac family small GTPase 1 (*RAC1*), mitogen-activated protein kinase 1 (*MAPK1*), mitogen-activated protein kinase kinase 1 (*MAP2K1*), and mitogen-activated protein kinase kinase 2 (*MAP2K2*).

### Estimation of SNV Frequency for Single Gene and Gene Set

The frequency of SNV for each gene in the RTK/RAS pathway was calculated. Seven types of deleterious mutations were included in this analysis: missense, nonsense, frameshift insertion, splice site, frameshift deletion, in-frame deletion, and in-frame insertion. GSCA, which represents the integrated SNV status of a gene set, was also used to perform gene set SNV analysis. Briefly, a sample was classified into the mutant group only when at least one gene in the RTK/RAS pathway was mutated in this sample. If none of the genes in the RTK/RAS pathway were mutated in a sample, this sample was classified into the WT group.

### Estimation of CNV Frequency and Gene Set CNV

The frequencies of homozygous deletion or amplification and heterozygous deletion or amplification were summarized. Gene set CNV was performed using GSCA. The gene set CNV represented the integrated CNV status of a gene set; here, it is the RTK/RAS pathway. A sample was classified into an amplification or deletion group only when at least one gene in the RTK/RAS pathway was consistently amplified or deleted in this sample. If all genes in the RTK/RAS pathway carried no CNV in a sample, the sample was classified into the WT group. The samples with both amplifications and deletions of genes in the RTK/RAS pathway were excluded from the gene set CNV analysis.

### Differential Methylation and Differential Expression Analysis

Differential methylation and expression analyses were performed for the RTK/RAS pathway genes in 29 and 58 paired tumor-normal LUAD samples, respectively. The methylation site that was most negatively correlated with mRNA expression was filtered for each gene in the methylation analysis (**Table S1**). The Wilcoxon rank sum test was performed to compare methylation and expression levels between tumor and normal samples. To control the false discovery rate (FDR), the P-value was adjusted using the multiple testing method: Benjamini & Hochberg. The difference in methylation (tumor-normal) and FDR was used to evaluate the change in methylation between the groups.

### Estimation of the Abundance of Immune Cells

ImmuCellAI (13) predicted the abundance of each immune cell tpe in each sample based on the mRNA expression data of immune cell markers. The abundance of 24 immune cells, including dendritic cells, B cells, monocytes, macrophages, natural killer cells (NK), neutrophils, CD4 T cells (CD4_T), CD8 T cells (CD8_T), natural killer T cells (NKT), gamma delta T cells (gamma_delta), CD4 naïve T cells (CD4_naive), CD8 naïve T cells (CD8_naive), cytotoxic T cells (cytotoxic), exhausted T cells (exhausted), type 1 regulatory T cells (Tr1), natural Tregs (nTregs), induced regulatory T cells (iTregs), T helper type 1 (Th1), T helper type 2 (Th2), T helper type 17 (Th17), T follicular helper cells (Tfh), central memory T cells (Central_memory), effector memory T cells (effector_memory), and mucosal-associated invariant T cells (MAIT) were estimated for each sample *via* ImmuCellAI (13).

### Survival Analysis

The OS, PFS, DSS, and DFI survival data and SNV, CNV, and methylation data were merged using sample barcodes. Tumor samples were grouped before analysis. For SNV data, tumor samples were divided into mutant and WT groups according to the SNV status of the gene. For CNV survival analysis, tumor samples were classified into three groups according to homozygous and heterozygous CNV status, including amplification, deletion, and WT (**Table S3**). Additionally, the pairwise_survdiff function in the survminer R package was used for the pairwise comparison among the three groups and correction of the P-value by using multiple testing. For methylation and expression data, tumor samples were divided into higherhigh and low groups through the middle values. R package survival was used to fit survival time and status. The Cox proportional hazards model and log-rank tests were performed to test the survival difference between more than two groups. R package survminer was used to generate the survival curves. Statistical significance was set at P-value ≤ 0.05.

### Construction of Nomograms

Mutation, clinical, and immune cell abundance data were combined using sample barcodes. The NAs in the data were removed. First, for each type of survival data, the samples were randomly separated into training (70%) and test (30%) sets. The R package of caret was used for random selection of samples. Second, according to the univariable survival analysis result, the variables dependently associated (Cox P-value < 0.05) with OS, PFS, DSS, and DFI were selected to construct multiple variable Cox proportional hazard models for each survival type. The variables were then ranked by Cox P-value significance. The most significant feature was added to the first model. For example, Model 1 had the most significant feature, Model 2 had the two most significant features, and so on. Third, the training set samples were used to perform 10 runs of 10-fold cross-validation. The rms R package was used to construct a nomogram based on a parametric survival model. Concordance index (C-index) was used to evaluate model performance. The best-performing model was selected on the basis of the average C-index of the 10 runs of 10-fold cross-validation. Fourth, the model was retrained with the entire training set and further tested in the test set to evaluate the model’s performance. The calibration of the nomograms was assessed graphically through bootstrap resampling validation.

### Statistical Analysis

Spearman’s rho statistic was used to estimate a rank-based measure of association. The Wilcoxon rank sum test was used for comparisons between two groups. Multiple testing was performed using the Benjamini & Hochberg method to control the FDR. Statistical significance was set at FDR ≤ 0.05. All statistical analyses in this study were performed using R language.

## Results

### Landscape of the RTK/RAS Pathway SNVs and its Relevance With mRNA Expression in LUAD

**Figure 1:**
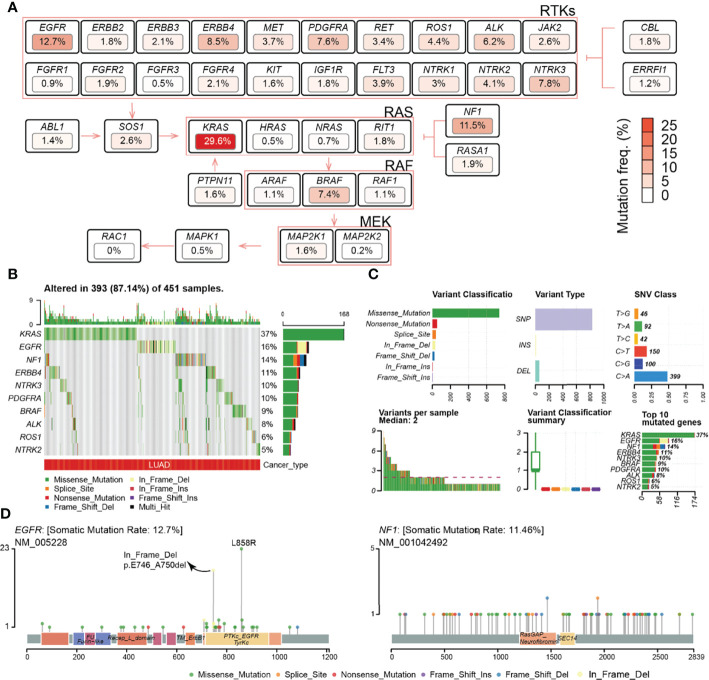
Frequency and class of the SNV of the RTK/RAS pathway. **(A)** Summary of the SNV frequency of the RTK/RAS pathway and the inner relationship within the signaling path. Color is correlated with the frequency of SNV. **(B)** Summary of the variant type and classification of RTK/RAS pathway genes. **(C)** Distribution of the SNV of top 10 frequently mutated genes in LUAD samples. **(D)** Lollipop plot showing the location and count of SNV in the coding region of EGFR and NF1.

The frequency of deleterious SNV in the RTK/RAS pathway in the TCGA LUAD samples was summarized. The results indicated that the deleterious mutation rates of the RTK/RAS pathway genes ranged from 0% to 29.6% (**Table S4**; **Figure 1A**). A total of 451 LUAD samples contained at least one mutation in the RTK/RAS pathway (**Figure 1B**). Of these samples, 393 (87.14%) had at least one mutation among the top 10 mutated genes (**Figure 1B**). *KRAS* mutations were detected in 37% of the 393 samples. *KRAS* (SNV: 29.6%) was the most frequently mutated gene in the RTK/RAS pathway, *EGFR* (also known as *ERBB1*, 12.7%), and *NF1* (11.5%) followed by *KRAS*. Downstream of the RTK/RAS pathway, *RAC1* was normally encoded without any deleterious SNV.

The mutation of genes within the RTK/RAS pathway, even within the ERBB, NTRK, RAF, and RAS families, showed heterogeneity. At the pathway level, *KRAS*, *EGFR*, and *NF1* were exclusively mutated, with only two samples containing both *KRAS* and *EGFR* mutations, 16 samples containing *KRAS* and *NF1* mutations, and two samples containing *EGFR* and *NF1* mutations (**Figure S1A**). At the gene family level, genes within the ERBB (**Figure S1B**), NTRK (**Figure S1B**), RAS (**Figure S1B**), and RAF (**Figure S1B**) families all showed exclusive mutations. In the ERBB family, for example, the SNV frequencies of *ERBB4* (8.5%) and *EGFR* (12.7%) were higher than those of *BRBB2* (1.8%) and *BRBB3* (2.1%), and only 9 of 133 samples with *ERBB4* and *EGFR* both mutated (**Figure S1B**). For RAF and NTRK families, the most frequently mutated genes were *BRAF* (7.4%) and *NTRK3* (7.8%), respectively. These results indicated that lung tumors would avoid the overloading of oncogenic signaling in the same pathway/family in the system through a mutually exclusive mutation mechanism.

The statistics of variant classification (**Figure 1C**) showed that most of the variants in the RTK/RAS pathway were missense mutations, and the most frequent variant type was single nucleotide polymorphism (SNP). Among the SNPs, the C > G variant was the most common SNV. The SNV types and locations of *KRAS* were concentrated (**Figure S2A**), whereas the SNV types and locations of *EGFR* and *NF1* were diverse (**Figure 1D**), especially for *NF1*, whose SNV locations were dispersed in its coding region. Interestingly, *EGFR* carried a high frequency of in-frame deletions (mainly in exon 19), specifically in LUAD (**Figure S2B**), and *EGFR* carried missense mutations in exon 21, while these two types of mutations encoded the TyrKc protein domain (**Figure 1D**).

Additionally, the effect of SNV on mRNA expression was explored (**Table S5**; **Figure S3A**). The results showed that the mutations on *KRAS*, *EGFR*, and *ABL1* were significantly associated with their higher expression levels (**Figure S3B**). Mutations in *NTRK3* and *ROS1* were significantly related to their low expression (**Figure S3B**). An insufficient sample size in the mutant group (e.g., *ABL1* and *RASA1*) might lead to low-level statistical significance.

### Summary of CNV of the RTK/RAS Pathway and its Relevance With mRNA Expression in LUAD

**Figure 2:**
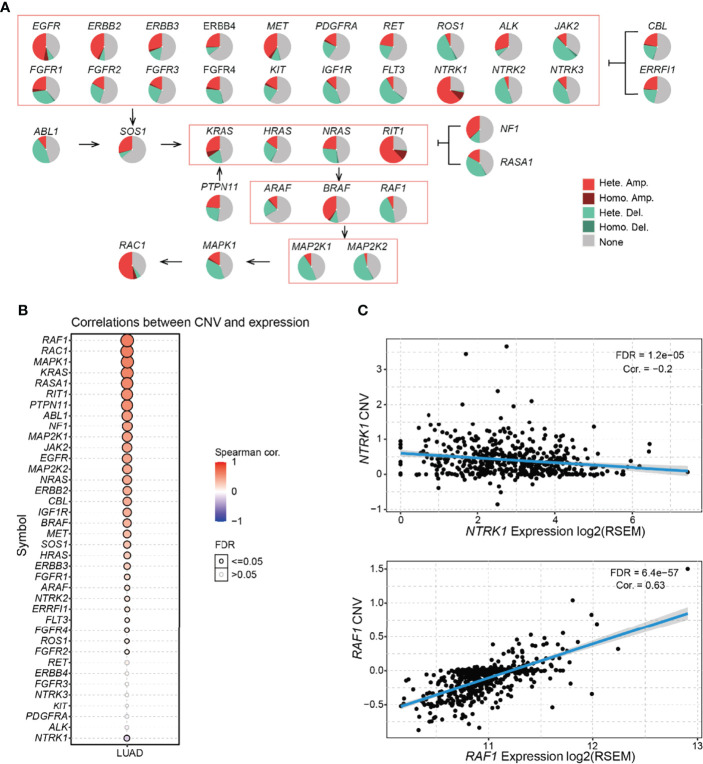
Frequency and class of CNV of the RTK/RAS pathway and its influence on mRNA expression. **(A)** Summary of the frequency of four CNV types in RTK/RAS pathway genes. Hete.: heterozygous; Homo.: homozygous; Amp.: amplification; Dele.: deletion. **(B)** Spearman correlation between the CNV and mRNA expression of RTK/RAS pathway genes. Blue and red bubbles represent negative and positive correlations, respectively. Bubble size is positively correlated with the FDR significance. The black outline borders indicate FDR ≤ 0.05. **(C)** The scatter plot, with a fitted line shows the detailed relationship between mRNA expression and CNV of NTRK1 and RAF1.

Most RTK/RAS pathway genes carried CNV in more than 50% of the LUAD samples (**Table S3**; **Figure 2A**). Among the RTK/RAS pathways, heterozygous CNV occurred frequently. However, only *EGFR*, *NTRK1*, *KRAS*, and *RIT1* carried homozygous CNV in > 5% of LUAD samples. In addition, the CNV status varied among genes. For example, *NTRK1*, *RIT1*, *RAC1*, *EGFR*, and *ERBB2* were mainly amplified, whereas *ROS1*, *JAK2*, *FLT3*, *MAPK*, *MAP2K1*, and *MAP2K2* were mainly deleted in LUAD.

mRNA expression is frequently associated with CNV status (14, 15). Gene amplification increases mRNA expression, deletion decreases mRNA expression. Here, we performed correlation analyses between the expression and CNV of RTK/RAS pathway genes in LUAD (**Figure 2B**). The results showed that the expression levels of 30 of 38 RTK/RAS pathway gene were positively correlated with their CNV level (FDR ≤ 0.05). For example, significantly upregulated genes, such as *MET* and *ERBB2* (**Figure S2**), were highly amplified and their expression was positively correlated with CNV. Although *NTRK1* was amplified in more than 70% of LUAD tumor samples, its mRNA expression was downregulated in LUAD tumor samples (**Figure S2**). Unsurprisingly, the mRNA expression of *NTRK1* was negatively correlated with its CNV level (**Figure 2C**). Our results suggested a strongly negative regulatory factor to counteract the high frequency of *NTRK1* amplification, eventually leading to *NTRK1* downregulation.

### Differential Methylation of RTK/RAS Pathway and its Correlation With mRNA Expression in LUAD

**Figure 3:**
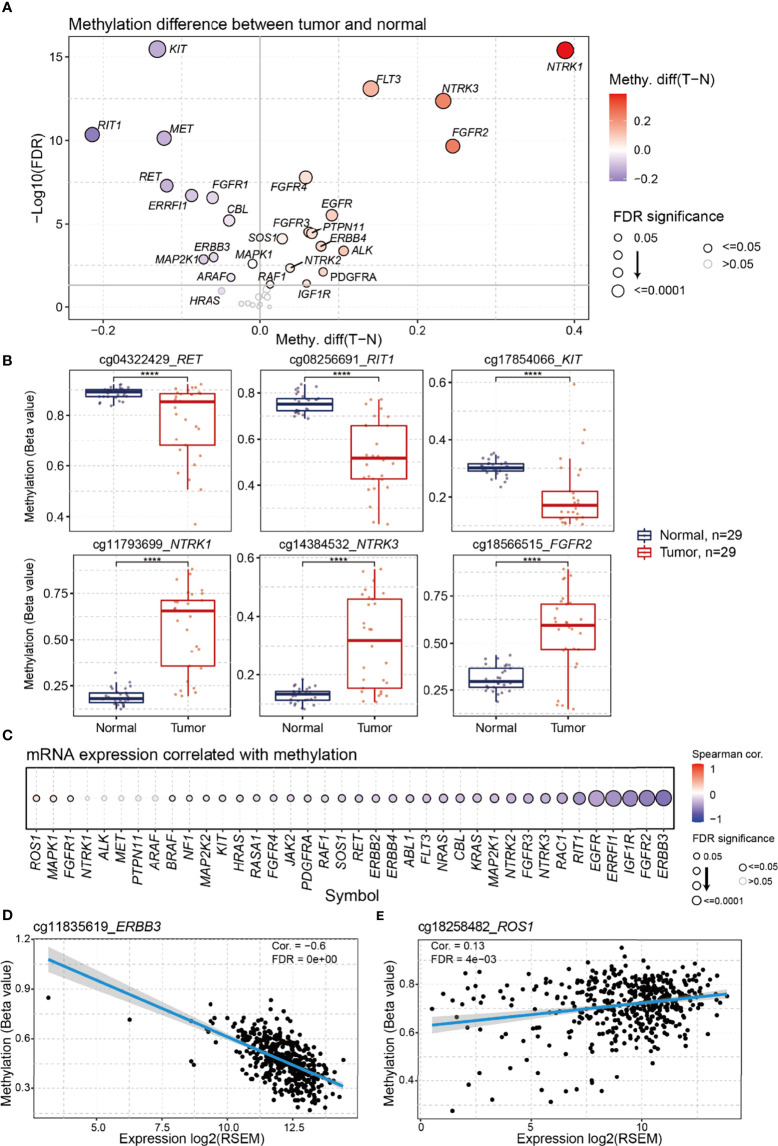
Differential methylation of RTK/RAS pathway and its correlation with mRNA expression. The methylation sites used in this analysis were showing in **Table S2**. **(A)** Methylation difference between tumors and normal samples. Blue and red bubbles represent a decrease and increase in methylation in tumors compared with that in normal samples, respectively. Bubble size is positively correlated with the FDR significance. **(B)** Boxplot showing the difference in *RET*, *RIT1*, *KIT*, *NTRK1*, *NTRK3*, and *FGFR2* methylation between tumors and normal samples. ****FDR < 0.0001. **(C)** Bubble plot summarizing the Spearman correlation between the methylation and mRNA expression of RTK/RAS pathway genes. Blue and red bubbles represent negative and positive correlations, respectively. Bubble size is positively correlated with the FDR significance. The black outline borders indicate FDR ≤ 0.05. Scatter plot with a fitted line showing the detailed relationship between the mRNA expression and methylation of **(D)** *ERBB3* and **(E)** *ROS1*.

DNA methylation is an important regulator of gene expression and function (16). We performed differential methylation analysis and found that 26 genes in the RTK/RAS pathway were differentially methylated (**Figure 3A**, **Table S6**). Among the 26 genes, *RIT1*, *KIT*, and *RET* were the most hypomethylated genes in LUAD, whereas *NTRK1*, *NTRK3*, and *FGFR2* were the most hypermethylated genes (**Figure 3B**).

To investigate the influence of DNA methylation on mRNA expression, we performed correlation analyses (**Figure 3C**). The results indicated that the expression levels of 30 RTK/RAS pathway genes, such as *ERBB3*, were negatively correlated with DNA methylation (**Figure 3D**). However, the mRNA expression levels of the three genes, namely, *ROS1* (**Figure 3E**), *MAPK1*, and *FGFR1*, were weakly and positively correlated with methylation. Combined with the differential expression results (**Figure S4**), our findings indicated that DNA hypermethylation negatively regulated the mRNA expression levels of *FGFR2*, *FGFR4*, *ERBB4*, and *NTRK3*. DNA hypomethylation could be a positive regulator of *RET* and *MET* expression. Unexpectedly, the mRNA expression levels of the most hypomethylated *KIT* and hypermethylated *NTRK1* were undifferentiated in the tumors.

### Survival Relevance of the RTK/RAS Pathway in LUAD

**Figure 4:**
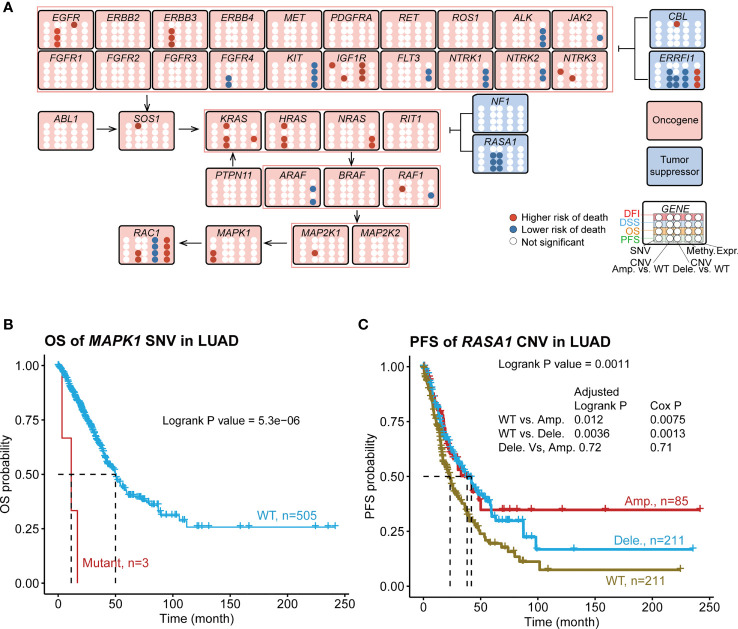
Associations of RTK/RAS pathway mutation with four survival types of LUAD. **(A)** Summary of the survival results of OS, PFS, DSS, and DFI of the SNV, CNV, methylation, and mRNA expression of RTK/RAS pathway genes. Blue and red points represent the low and higherhigh risks of death for SNV/CNV/high methylation/high expression groups, respectively. For CNV, Amp. vs. WT and Dele. vs. WT were compared, respectively. **(B)** K–M survival curves showing the OS difference between *MAPK1* SNV groups. **(C)** PFS difference between *RASA1* CNV groups. Pair wise comparison was performed, and P value was adjusted by FDR.

We investigated the survival relevance of genomic changes (**Figure 4A**; **Table S7A**). We found that several rarely mutated genes were significantly associated with the rapid death and progression of patients with LUAD. For example, mutant *MAPK1* (n=3) was associated with shorter OS (**Figure 4B**) and PFS (**Figure S5A**) at 10–20 months than WT; patients who had these mutants progressed and died rapidly. The mutant *IGF1R* (n=4) showed a similar phenomenon in DFI (**Figure S5B**). However, the sample size difference was too large between the WT and mutant groups. Thus, we randomly selected a subset sample (2% and 5%) from WT to generate a WT group with a smaller sample size, which was called “mini-WT,” and we performed survival analyses between the mini-WT and mutant groups. This process was repeated 100 times for each sample size (2% and 5%). The results (**Table S8**; **Figure S6A**) showed that the survival rate and median survival time of the mutant group were worse than those of the mini-WT group. And average of 87% (**Table S8**) of these repeats confirmed that patients who suffered from mutations in *MAPK1* and *IGF1R* had worse survival status than WT. The K-M survival curves of several iterations are shown in **Figure S6B**. In addition, the moderately mutated *NTRK3* (n=37) was associated with lower OS and DSS rates and shorter median survival time (**Figure S5C**). However, highly mutated genes such as *EGFR* and *KRAS* were weakly associated with LUAD survival.

We found that the mutation status of the exclusively mutated *KRAS* and *NF1* was associated with PFS and DFI (**Figure S7A**). Compared with WT, *KRAS*- or *NF1*-mutated samples, the co-occurrence of *KRAS* and *NF1* mutations led to worse PFS and DFI (**Figure S7B**). The *KRAS*–*NF1* co-mutated group had approximately 16 samples, and they were far from the *KRAS* and WT groups. To avoid contingency, we randomly selected 10% of the samples from the other groups and performed 100 iterations (**Figure S7C**). The results showed that average of 78% (**Table S8**) of iterations confirmed that the survival rate and median survival time of the *KRAS*–*NF1* co-mutated group were worse than those of the other mini-groups, including mini-*KRAS* (**Figure S7D**) and mini-WT (**Figure S7D**). This result confirmed that patients who had LUAD and mutated *KRAS* and *NF1* had worse PFS and DFI.

To find the biological interpretation of the phenomenon that *KRAS–NF1* co-mutation is relevant to worse survival, we performed functional analysis. *KRAS–NF1* co-mutated and WT LUAD samples were selected for differential expression analysis. The results indicated that 138 genes were dysregulated (FDR < 0.05) in *KRAS–NF1* co-mutated LUAD samples, including 72 downregulated genes and 66 upregulated genes (**Table S9**, **Figure S8A**). Kyoto Encyclopedia of Genes and Genomes (KEGG) pathway gene set enrichment analysis showed that numerous genes in the ribosomal pathway were downregulated (**Table S9**; **Figure S8B**), indicating insufficient protein synthesis in patients with *KRAS–NF1* co-mutated LUAD. Additionally, the cAMP signaling pathway was associated with extensive intracellular signal transduction. The downregulation of the cAMP signaling pathway (**Figure S8B**) indicated intracellular signal deactivation in *KRAS–NF1* co-mutated patients. The enrichment in the chemical carcinogenesis KEGG pathway (**Figure S8B**), combined with the enrichment in immune response Gene ontology biological process (**Figure S8C**), suggested the possibility of environmental factors causing *KRAS–NF1* co-mutation. We further focused on changes in the mRNA expression of genes downstream of the RTK/RAS pathway (**Figure S8D**). Among the 22 RTK/RAS downstream genes (**Table S10**), only phospholipase A2 group IVB (*PLA2G3*) was significantly downregulated in patients with *KRAS–NF1* co-mutation. Therefore, the effect of *KRAS–NF1* co-mutation on the mRNA expression of downstream receptors was minimal.

The overall survival comparison showed that the survival of three CNV groups (WT, amplification, and deletion) of *EGFR*, *ERBB3*, *FGFR4*, *IGF1R*, *NTRK3*, *RAF1*, *RASA1*, and *ROS1* were significantly differed (**Table S7B**). To investigate the difference in survival between each two groups, we performed a pairwise comparison between the three groups (**Figure 4A**; **Table S7C**). The results of the pairwise comparison were consistent with the overall survival comparison (**Table S7B**), with at least one of these three comparisons (WT vs. Amp., WT vs. Dele., Dele. Vs Amp.) indicating a significant difference in survival. The amplification or deletion of these genes was consistently associated with a higher risk of death except for the oncogene *FGFR4* and the tumor suppressor genes *ERRFI1* and *RASA1* (**Figure 4C**; **Figure S5D**). We also found 17 new survival relevancies (**Table S7C**) in pair wise comparison. For example, the Cox proportional hazard model indicated that the amplifications of *ERBB3*, *RAC1*, *KRAS*, *HRAS*, and *MAP2K1* were related to worse OS than those of WT; the amplification of *HRAS*, *RAC1*, and *KRAS* were associated with worse PFS; the amplification of *HRAS* and *KRAS* and the deletion of *CBL* were relevant to worse DFI; the amplification of *ERRFI1* was related to better DFI; and the amplification of *ERBB3* was related to worse DSS (**Table S7C**). The deletion and amplification of *RASA1* (**Figure 4C**; **Figure S5D**) and *FGFR4* (**Table S7C**) were consistently associated with better survival. For *NTRK3*, *IGF1R*, and *RAF1*, the survival status differed between the amplification and deletion groups (**Table S7C**). For instance, patients with *RAF1* amplification showed a lower DSS survival rate and shorter middle survival time than those with *RAF1* WT and deletion did (**Figure S5E**).

The methylation levels of *IGF1R*, *ERRFI1*, and *RAC1* were significantly associated with the survival of patients with LUAD (**Figure 4A**; **Table S7D**). Among them, the high methylation of *IGF1R* and *EGFR* served as a poor prognostic indicator (high risk of death in the high methylation group), whereas the high methylation of *ERRFI1* and *RAC1* served as a good prognostic predictor (lower risk of death in the high methylation group). Among these genes, *EGFR* methylation was only relevant to DFI survival, while the others were relevant to more than three types of survival.

mRNA expression is influenced by genomic status. Thus, we checked the survival relevance of the mRNA expression of the RTK/RAS pathway genes. The results indicated that the expression levels of 12 genes were relevant to LUAD survival (**Figure 4A**), four of which served as poor prognostic predictors (high risk of death in the high expression group), while the others served as good prognostic predictors (lower risk of death in the high expression group). Among these genes, the expression levels of *KRAS*, *RAF1*, and *JAK2* were associated only with OS, while others were associated with more than two types of survival time.

Combining the survival results of SNV, CNV, methylation, and mRNA expression of the RTK/RAS pathway, we found that only six genes, namely, *EGFR*, *IGF1R*, *NTRK3*, *ERRFI1*, *RAF1*, and *RAC1*, with multiple types of genomic changes were associated with survival were associated with survival (**Figure 4A**). Among them, the high expression of *RAC1* or *ERRFI1* was associated with a high risk of death, while high methylation was associated with a lower risk of death. This result was consistent with the negative correlation between the mRNA expression and methylation of *RAC1* and *ERRFI1*.

### Relationship Between Mutation and Immune Cell Abundance

**Figure 5:**
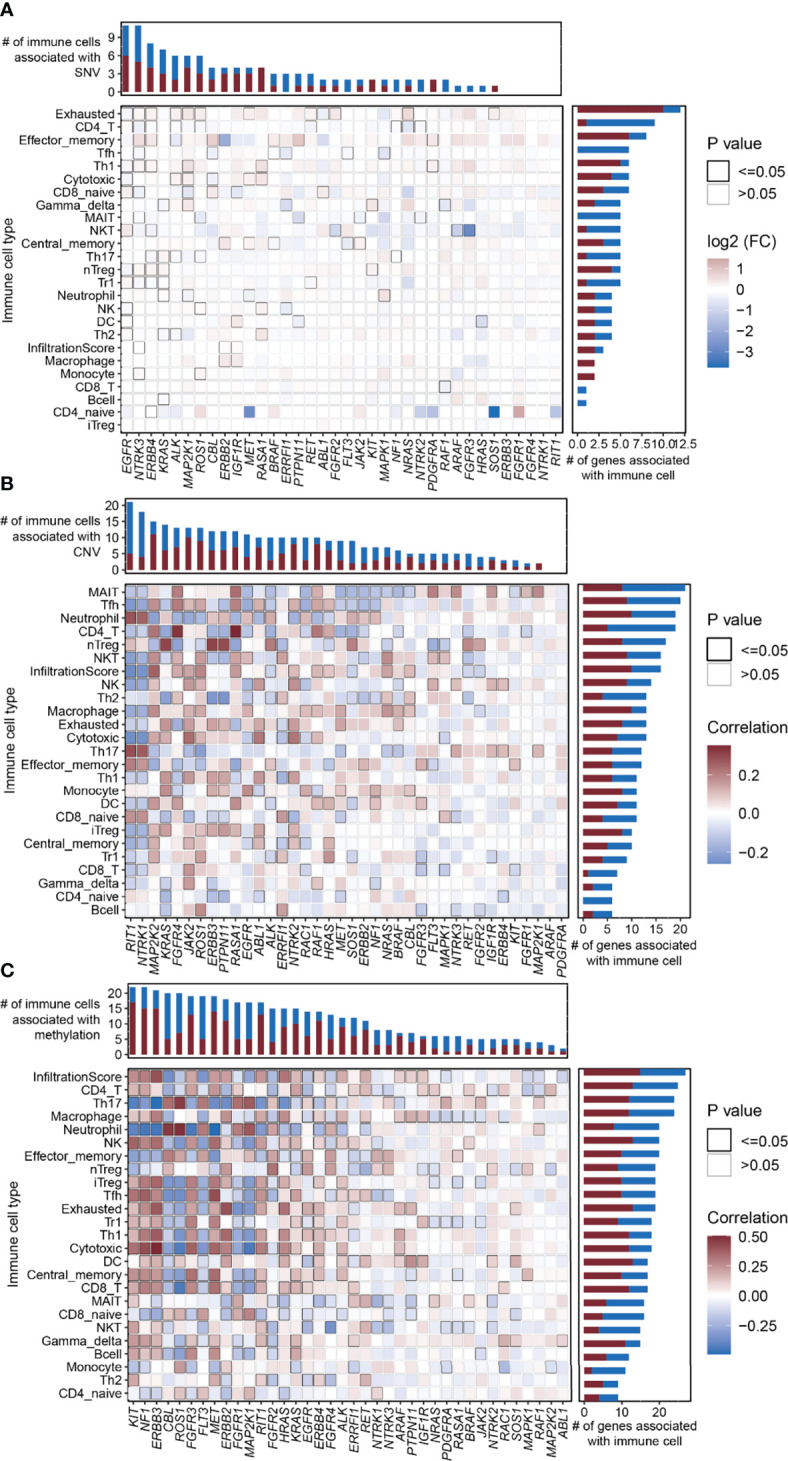
Correlation between the RTK/RAS pathway mutation and abundance of 24 immune cells in LUAD. **(A–C)** Heatmaps showing Spearman correlation between 24 immune cells/overall infiltration score of 24 immune cells and the **(B)** SNV, **(C)** CNV, and **(D)** methylation of RTK/RAS pathway genes. The color of each cell is correlated with the fold change or correlation coefficient. The black outline border of each cell indicates P ≤ 0.05.

The abundance of 24 immune cell types was estimated using ImmuCellAI (13). We analyzed the relationship between the SNV/CNV/methylation of the RTK/RAS pathway genes and the abundance of 24 immune cells (**Table S11**). At the single gene level, the SNV of the top mutated genes, including *EGFR* (frequency of SNV: 12.7%), *NTRK3* (7.8%), *ERBB4* (8.5%), and *KRAS* (29.6%), were correlated with the abundance of 11, 11, 9, and 7 immune cell types, respectively. The SNVs of *RIT1*, *NTRK1*, *FGFR4*, *FGFR1*, and *ERBB3* were not associated with immune cell abundance (**Figure 5A**). The increased abundance of exhausted cells was related to SNVs of the 10 RTK/RAS pathway genes (**Figure 5A**). In addition, the decreased abundance of CD4 T and Tfh cells was associated with the SNVs of eight and six RTK/RAS pathway genes, respectively (**Figure 5A**). The gene set level analysis also supported that the SNV gene set of the RTK/RAS pathway was associated with the activation of nTregs, neutrophils, and Th17 and the inactivation of gamma delta, MAIT, and Tfh (**Figure S9A**).

At the single gene level, the copy number of highly amplified genes, including *RIT1* (frequency of amplification: 73%), *NTRK1* (72%), and *MAP2K2* (53%), were correlated with the greatest number of immune cell types, i.e., 21, 18, and 15, respectively (**Figure 5B**; **Table S11**). Among them, the CNVs of *RIT1* and *NTRK1* were associated with immune cell inhibition, whereas the CNV of *MAP2K2* was correlated with immune cell activation (**Figure 5B**). Moreover, the overall copy number amplification status of the RTK/RAS pathway was associated with high activity of nTregs and low activity of NKT and cytotoxicity. The overall copy number deletion status of the RTK/RAS pathway was associated with the inactivation of macrophages, MAIT, NKT, and NK and the activation of Th17 and CD8 naive cells (**Figure S9B**).

Correlation analysis between the methylation of RTK/RAS pathway genes and the abundance of 24 immune cell types (**Figure 5C**; **Table S11**) showed that *KIT*, *NF1*, *ERBB3*, *FGFR3*, *MET*, *ERBB2*, *RIT1*, *HRAS*, *KRAS*, *ERBB4*, *ALK*, *RET*, *ARAF*, *PTPN11*, and *IGF1R* were positively correlated with immune cells. *CBL*, *ROS1*, *FLT3*, *FGFR1*, *MAP2K1*, *FGFR2*, and *PDGFRA* were negatively correlated with immune cells.

In summary, the higher the mutation rate of a gene, the closer the relationship between the gene and immune cell abundance. For example, in comparison with other genes, genes with high SNV frequencies, such as *EGFR* (n=11 immune cell types), *NTRK3* (n=11), ERBB4 (n=9), and *KRAS*; genes with high CNV rates, such as *RIT1* and *NTRK1*; and genes with high methylation differences between tumor and normal tissues, such as *KIT*, were correlated with the increased immune cell abundance.

### Nomogram for Predicting the OS, PFS, DSS, and DFI Probability of LUAD

Immune cell abundance in TME is closely associated with survival (17). To investigate the survival relevance of immune cell abundance, we performed survival analyses to determine the abundance of 24 immune cells in LUAD. The results indicated that the high abundance of monocytes and neutrophils in the TME was associated with a high risk of death, while the high abundance of immune cells such as gamma_delta, CD8_T, and Tr1 was associated with a lower risk of death (**Figure S10A**; **Table S7E**). We found that mutations (SNV/CNV/methylation) in RTK/RAS pathway genes were potentially associated with patient survival (OS, PFS, DSS, and DFI) and immune cell abundance in the TME of LUAD. Thus, we integrated the genomic changes in the RTK/RAS pathway and immune cell abundance to construct a nomogram survival model for OS, PFS, DSS, and DFI of patients with LUAD. Although *MAPK1* and *IGF1R* were associated with survival, they were rarely mutated and had limited samples for cross-validation. Thus, the SNVs of *MAPK1* and *IGF1R* were not included in the construction of nomogram survival model.

**Figure 6:**
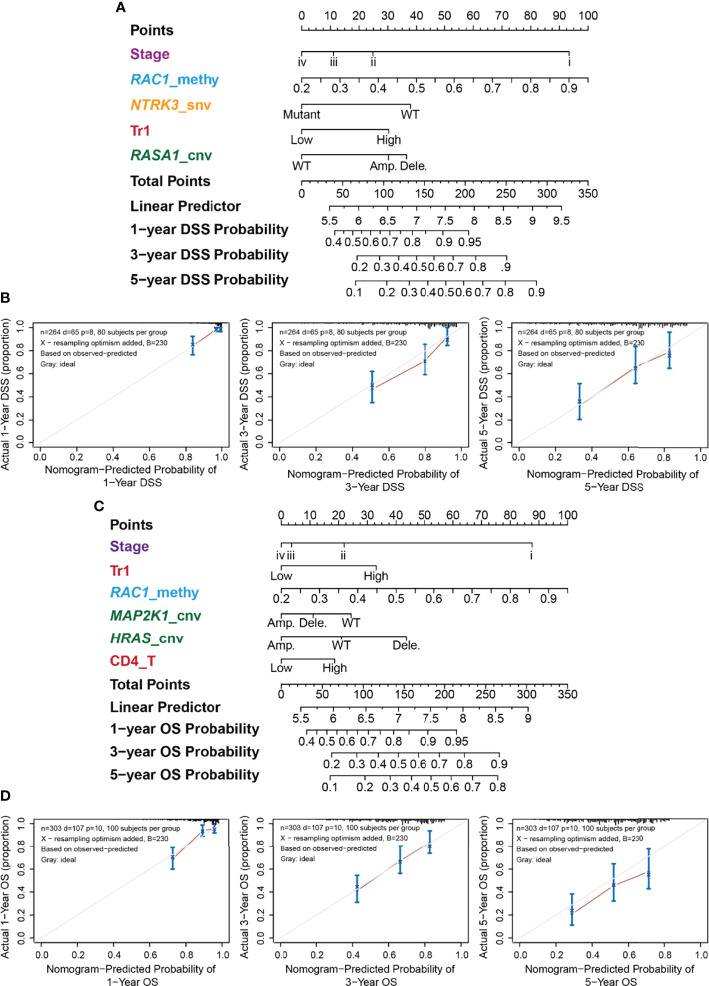
Nomogram for the DSS of LUAD and its performance. **(A)** Predictive nomogram of DSS for LUAD. The row “Points” correspond to each signature. For example, stage iv got 0 points, while stage i got nearly 90 points. The sum of signatures’ points is the row “Total Points,” which correspond to survival probability. **(B)** Correspondence between actual and ideal nomogram predictions for 1-, 3-, and 5-year DSS. Nomogram-predicted probability of survival is plotted on the x-axis; actual survival is plotted on the y-axis. **(C)** Predictive nomogram of OS for LUAD. **(D)** Correspondence between actual and ideal nomogram predictions for 1-, 3-, and 5-year OS.

For each survival type, genomic changes in the RTK/RAS pathway genes and immune cells that passed the univariate Cox proportional hazard analysis (**Table S7**) were selected to construct multivariate Cox proportional hazard models (**Table S12**). Then, the features were ranked in terms of Cox P-value significance to construct different nomogram models. Cross-validation was performed to screen features that performed well in predicting survival. The results (**Table S13**; **Figure S10B**) indicated that genomic changes in RTK/RAS and immune cell abundance performed well in predicting DSS and OS, but they were barely satisfactory in predicting DFI and PFS. The selected variables for OS, PFS, DSS, and DFI nomogram were Stage+Tr1+*RAC1*_methy+*MAP2K1*_cnv+*HRAS*_cnv+CD4_T, Stage+Tr1+*RAC1*_methy, *EGFR*_methy+*ERRFI1*_cnv+Stage+Gamma_delta+Age, and Stage+*RAC1*_methy+*NTRK3*_snv+Tr1+*RASA1*_cnv, respectively (**Table S13**; **Figure S10B**). The nomograms constructed for LUAD DSS (**Figure 6A**) and OS (**Figure 6C**) are shown. Tumor stage and *RAC1* methylation contributed the most to DSS prediction (**Figure 6A**), with a wide range of points. The model showed that patients with lower tumor stage, higher *RAC1* methylation levels, WT *NTRK3*, higher Tr cell infiltration in the TME, and *RASA1* CNV survived better than other patients with opposite status. The OS nomogram model (**Figure 6C**) shared the predictors of tumor stage, *RAC1* methylation, and Tr cell infiltration of DSS. *MAP2K1* CNV, *HRAS* CNV, and CD4 T cell infiltration were other OS predictors.

The performance of the nomograms was evaluated using the C-index, which indicates the probability that the predicted results are consistent with the actual observed results. The C-indices of the nomogram in the training and test sets were 0.728 (95% CI 0.67–0.785, P < 0.001) and 0.689 (0.603–0.775, P < 0.001) for OS, and 0.771 (0.71–0.832, P < 0.001) and 0.7 (0.589–0.811, P < 0.001) for DSS, respectively. The performances of the nomograms of DSS (**Figure 6B**) and OS (**Figure 6D**) were also evaluated using calibration plots. The line segment was close to 45°, indicating good correspondence between the actual and ideal nomogram predictions, especially for 1- and 5-year DSS (**Figure 6C**) and 3-year OS (**Figure 6D**).

## Discussion

Genomic changes deeply influence molecular processes, leading to cancer development. The disturbance of the RTK/RAS pathway is associated with excessive proliferation (18); however, its association with immune infiltration in the TME and patient survival is only partially known. In this study, we summarized the mutation landscape (SNV, CNV, and DNA methylation) of the RTK/RAS pathway in LUAD, its relevance to mRNA expression, four survival types, and immune cell abundance. Moreover, we integrated the mutation profile of the RTK/RAS pathway, clinical features, and immune cell abundance to construct nomograms for the four survival types. We found that tumor stage, Tr1 infiltration, and *RAC1* methylation were important for DSS and OS nomograms.

Rare mutations are functionally important in cancer (19). We found that rarely mutated genes *MAPK1* and *IGF1R* were associated with OS or PFS and DFI, respectively. These results revealed the potential effect of rarely mutated genes on survival. However, because of the low frequency of these mutations, future studies will need more samples to validate the prognostic effect of these rarely somatic mutated genes. Frequently mutated genes are more likely to be driver mutations in cancer initiation and dissemination (20), and they are associated with clinical outcomes. In our study, although the frequently mutated genes among RTK/RAS were not associated with any survival types. And patients with co-mutation of *KRAS* and *NF1* had worse survival, and its underlying mechanism could be insufficient for protein synthesis and intracellular signal deactivation. Moreover, frequently variable genes were involved in immune cell activities, including the SNV of *EGFR* (frequency of SNV: 12.7%), *NTRK3* (7.8%), *ERBB4* (8.5%), and *KRAS* (29.6%); the CNV of *RIT1* (frequency of amplification: 73%), *NTRK1* (72%), and *MAP2K2* (53%); and the methylation of *KIT*. This phenomenon reminds us of the potential effect of rarely mutated genes on survival prediction and the underlying regulation of frequently mutated genes on immune infiltration in the TME.

Immunity in the TME is closely related to immunotherapy and traditional treatment efficacy and patient prognosis (21–23). We found that the SNVs of *NTRK3*, *ERBB4*, *ALK*, *ROS1*, and *NRAS* in the RTK/RAS pathway were associated with a high abundance of exhausted T cells and lower abundance of CD4 T cells in the TME of LUAD. Combined with previous results showing that immune cell infiltration in LUAD is significantly lower than that in normal samples (20), our results indicated that the SNV of RTK/RAS pathway genes in LUAD would lead to CD4 T cell exhaustion and further result in cancer immune evasion.

## Conclusion

In summary, we integrated SNV, CNV, methylation, immune cell abundance, and clinical data to present the mutation landscape of the RTK/RAS pathway and its relevance to the four types of survival and immunity of the TME. We revealed two rarely mutated genes as potential prognostic predictors of LUAD. Co-mutation of *KRAS* and *NF1* served as poor survival predictor. They could provide new targets for precision medicine and reveal the potential power of rarely mutated genes in predicting patient survival. Our findings revealed inner associations between genomic changes of RTK/RAS pathway and TME immune infiltration. These associations help elucidate the mechanisms of immune disturbance in the TME and provide the possibility for the combination of targeted therapy and immunotherapy. In addition, the nomograms illustrated the probability for evaluating survival probability in a mutation-immune combination pattern.

## Data Availability Statement

The data supporting the results of this study are available from University of California Santa Cruz Xena (http://xena.ucsc.edu/) and GSCA (http://bioinfo.life.hust.edu.cn/GSCA/#/download-page) databases.

## Author Contributions

All authors worked collectively to complete this study. X-QY, LX, and MZ designed the study and write the first draft of the manuscript; X-QY, X-HY, Y-QY, and MZ performed the data analysis and result interpretation. LX and MZ reviewed the manuscript and provided suggestions. All authors have read and approved the final manuscript.

## Conflict of Interest

The authors declare that the research was conducted in the absence of any commercial or financial relationships that could be construed as a potential conflict of interest.

## Publisher’s Note

All claims expressed in this article are solely those of the authors and do not necessarily represent those of their affiliated organizations, or those of the publisher, the editors and the reviewers. Any product that may be evaluated in this article, or claim that may be made by its manufacturer, is not guaranteed or endorsed by the publisher.

## Supplementary Material

Supplementary Table 1 Methylation sites that analyzed in this study.

Supplementary Table 2 The sample size of each data type of LUAD in this study.

Supplementary Table 3 Summary of the CNV of 38 RTK/RAS pathway genes in LUAD.

Supplementary Table 4 Summary of the SNV of 38 RTK/RAS pathway genes in LUAD.

Supplementary Table 5 The mRNA expression difference between SNV and WT.

Supplementary Table 6 The methylation difference between LUAD and normal samples.

Supplementary Table 7 The result of OS, PFS, DSS, and DFI survival analysis for SNV, CNV, methylation, and immune infiltration.

Supplementary Table 8 The survival results of 100 iterations in randomly selected WT compared with rare mutated genes.

Supplementary Table 9 The differential expressed genes in *KRAS*-*NF1* co-mutated LUAD samples, and its pathway enrichment results.

Supplementary Table 10 22 RTK/RAS downstream genes.

Supplementary Table 11 The association of SNV, CNV, and methylation with immune cell abundance.

Supplementary Table 12 Multivariate Cox Proportional-Hazards models’ result.

Supplementary Table 13 10-fold cross validation of nomogram models.

## Glossary

**Table:**

ABL1	ABL proto-oncogene 1, non-receptor tyrosine kinase
ALK	ALK receptor tyrosine kinase
Amp	Amplification
ARAF	A-Raf protooncogene, serine/threonine kinase
BRAF	B-Raf proto-oncogene, serine/threonine kinase
CBL	Cbl proto-oncogene
CD4_naive	CD4 naïve T cells
CD4_T	CD4 T cells
CD8_naive	CD8 naïve T cells
CD8_T	CD8 T cells
Central_memory	central memory T cells
C-index	Concordance index
CNV	copy number variation
cytotoxic	cytotoxic T cells
Dele.	deletion
DFI	diseasefree survival
DSS	disease-specific survival
effector_memory	effector memory T cel l s
EGFR	epidermal growth f actor recepto r
ERBB2	erbb2 receptor tyrosine kinase 2
ERBB3	erb-b2 receptor tyrosine kinase 3
ERBB4	erb-b2 receptor tyrosine kinase 4
ERRFI1	ERBB receptor feedback inhibitor 1
exhausted	exhausted T cells
FDR	false discovery rate
FGFR1	fibroblast growth factor receptor 1
FGFR2	fibroblast growth factor receptor 2
FGFR 3	fibroblast growth factor receptor 3
FGFR 4	fibroblast growth factor receptor 4
FLT3	fms related receptor tyrosine kinase 3
gamma_delta	gamma delta T cells
HRAS	HRas proto-oncogene GTPase
IGF1R	insulin like growth factor 1 receptor
iTregs	induced regulatory T cells
JAK2	Janus kinase 2
KIT	KIT proto-oncogene, receptor tyrosine kinase
KRAS	KRAS proto-oncogene GTPase
LUAD	lung adenocarcinoma
MAIT	mucosalassociated invariant T cells
MAP2K1	mitogen-activated protein kinase kinase 1
MAP2K2	mitogen-activated protein kinase kinase 2
MAPK1	mitogenactivated protein kinase 1
MET	MET proto-oncogene, receptor tyrosine kinase
NF1	neurofibromin 1
NK	natural killer cells
NKT	natural killer T cells
NRAS	NRAS proto-oncogene GTPase
NSCLC	Non-small-cell lung cancer
nTregs	natural regulatory T cells
NTRK1	neurotrophic receptor tyrosine kinase 1
NTRK2	neuro t r op h i c r e c e p t o r ty r o s i n e ki n a s e 2
NTRK3	neurotrophic receptor tyrosine kinase 3
OS	overall survival
PDGFRA	platelet derived growth factor receptor alpha
PFS	progression-free survival
P L A 2 G 3	p h o s p h o l i p a s e A 2 g r o u p I V B
P T P N 1 1	p r o te i n ty r o si n e phospha ta se non-r ec e p tor type 11
RAC1	Rac family small GTPase 1
RAF1	Raf-1 proto-oncogene serine/ threonine kinase
RASA1	RAS p21 protein activator 1
RET	ret protooncogene
RIT1	Ras l ike wi thout CAAX 1
ROS1	ROS protooncogene 1 receptor tyrosine kinase
SNP	single nucleotide polymorphism
S N V	s i n g l e n u c l e o t i d e v a r i a t i o n
S O S 1	S O S R a s / Rac guanine nucleotide exchange factor 1
Tfh	T follicular helper cells
Th1	T helper type 1
Th17	T helper type 17
Th2	T helper type 2
TIL	tumor-infiltrating lymphocytes
TME	tumor microenvironment
Tr1	type 1 regulatory T cells
WT	wild type
