# Supplementary material for: Genomic Landscape of RTK/RAS Pathway and Tumor Immune Infiltration as Prognostic Indicator of Lung Adenocarcinoma
Source: Front Oncol. 2022 Jul 21;12:924239. doi: 10.3389/fonc.2022.924239 (PMC9351312; doi:10.3389/fonc.2022.924239)
Supplement: Supplementary Table 2 — The sample size of each data type of LUAD in this study. [file Table_2.docx]

**Table S2. The** **sample size of each data type of LUAD in this study.**

| SNV data | 567 LUAD samples that had SNV data. | 451 LUAD samples that hold at least one mutation from the RTK/RAS pathway. |
| --- | --- | --- |
| CNV data | 516 LUAD samples that had CNV data. |  |
| Methylation data | 29 LUAD samples with paired adjacent normal samples. | 525 samples in total. |
| mRNA expression data | 56 LUAD samples with paired adjacent normal samples. | 576 samples in total. |
