# Supplementary material for: Genomic Landscape of RTK/RAS Pathway and Tumor Immune Infiltration as Prognostic Indicator of Lung Adenocarcinoma
Source: Front Oncol. 2022 Jul 21;12:924239. doi: 10.3389/fonc.2022.924239 (PMC9351312; doi:10.3389/fonc.2022.924239)
Supplement: Supplementary Table 10 — 22 RTK/RAS downstream genes. [file Table_10.docx]

**Table S10. 22 RTK/RAS downstream genes.**

| Symbol | Full name |
| --- | --- |
| ELK1 | ETS transcription factor ELK1 |
| JMJD7-PLA2G4B | JMJD7-PLA2G4B readthrough |
| MYC | MYC proto-oncogene, bHLH transcription factor |
| PLA1A | phospholipase A1 member A |
| PLA2G10 | phospholipase A2 group X |
| PLA2G12A | phospholipase A2 group XIIA |
| PLA2G12B | phospholipase A2 group XIIB |
| PLAAT3 | phospholipase A and acyltransferase 3 |
| PLA2G1B | phospholipase A2 group IB |
| PLA2G2A | phospholipase A2 group IIA |
| PLA2G2C | phospholipase A2 group IIC |
| PLA2G2D | phospholipase A2 group IID |
| PLA2G2E | phospholipase A2 group IIE |
| PLA2G2F | phospholipase A2 group IIF |
| PLA2G3 | phospholipase A2 group III |
| PLA2G4A | phospholipase A2 group IVA |
| PLA2G4C | phospholipase A2 group IVC |
| PLA2G4D | phospholipase A2 group IVD |
| PLA2G4E | phospholipase A2 group IVE |
| PLA2G4F | phospholipase A2 group IVF |
| PLA2G5 | phospholipase A2 group V |
| PLA2G6 | phospholipase A2 group VI |
