# Supplementary material for: Genomic Landscape of RTK/RAS Pathway and Tumor Immune Infiltration as Prognostic Indicator of Lung Adenocarcinoma
Source: Front Oncol. 2022 Jul 21;12:924239. doi: 10.3389/fonc.2022.924239 (PMC9351312; doi:10.3389/fonc.2022.924239)
Supplement: Supplementary file 14 [file DataSheet_1.docx]

Supplementary materials


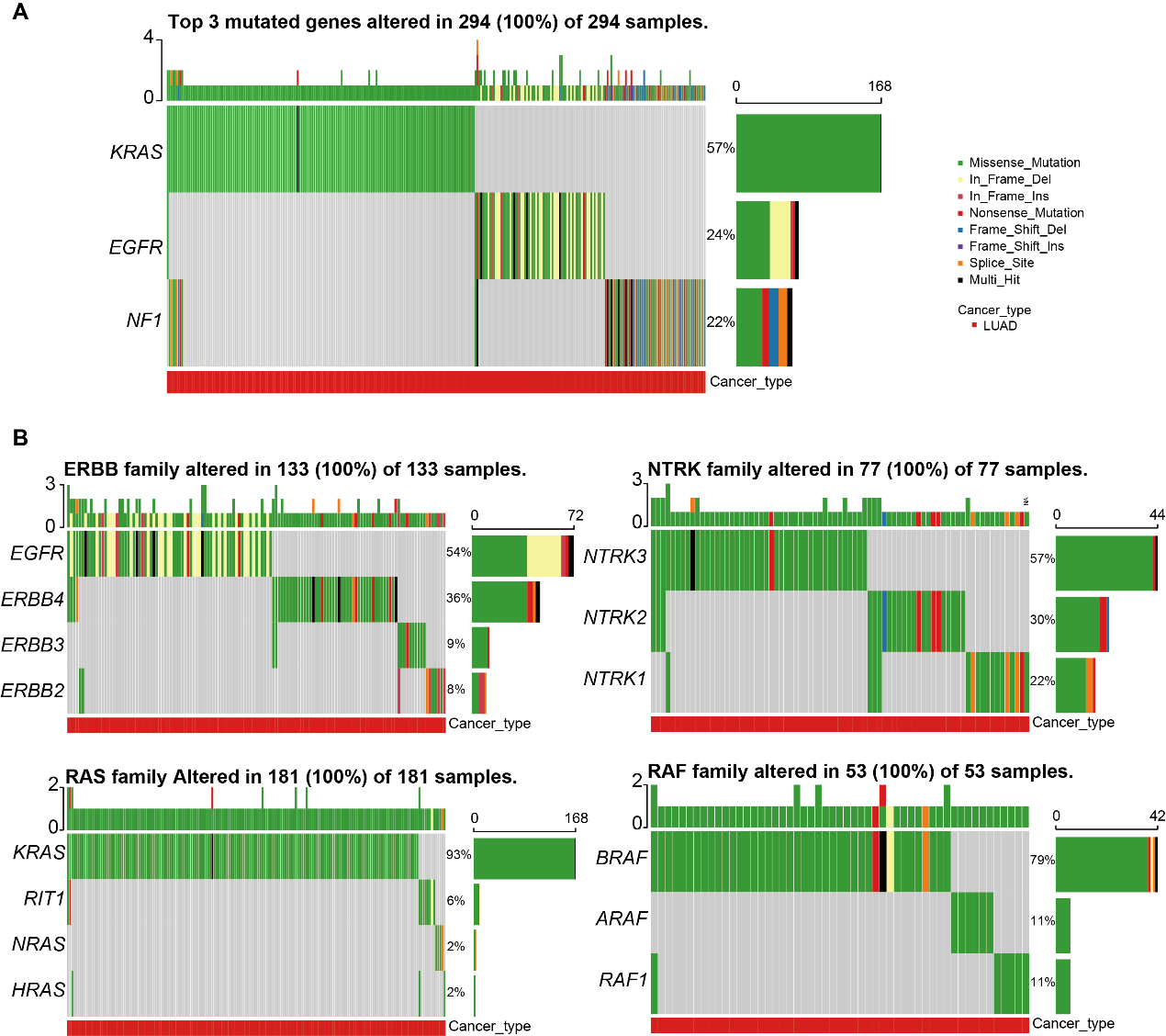


**Figure S1. Distribution of exclusive mutated genes in LUAD.** SNV distribution of (A) top 3 mutated genes (*KRAS*, *EGFR*, and *NF1*); (B) genes in ERBB, NTRK, RAS, and RAF family.


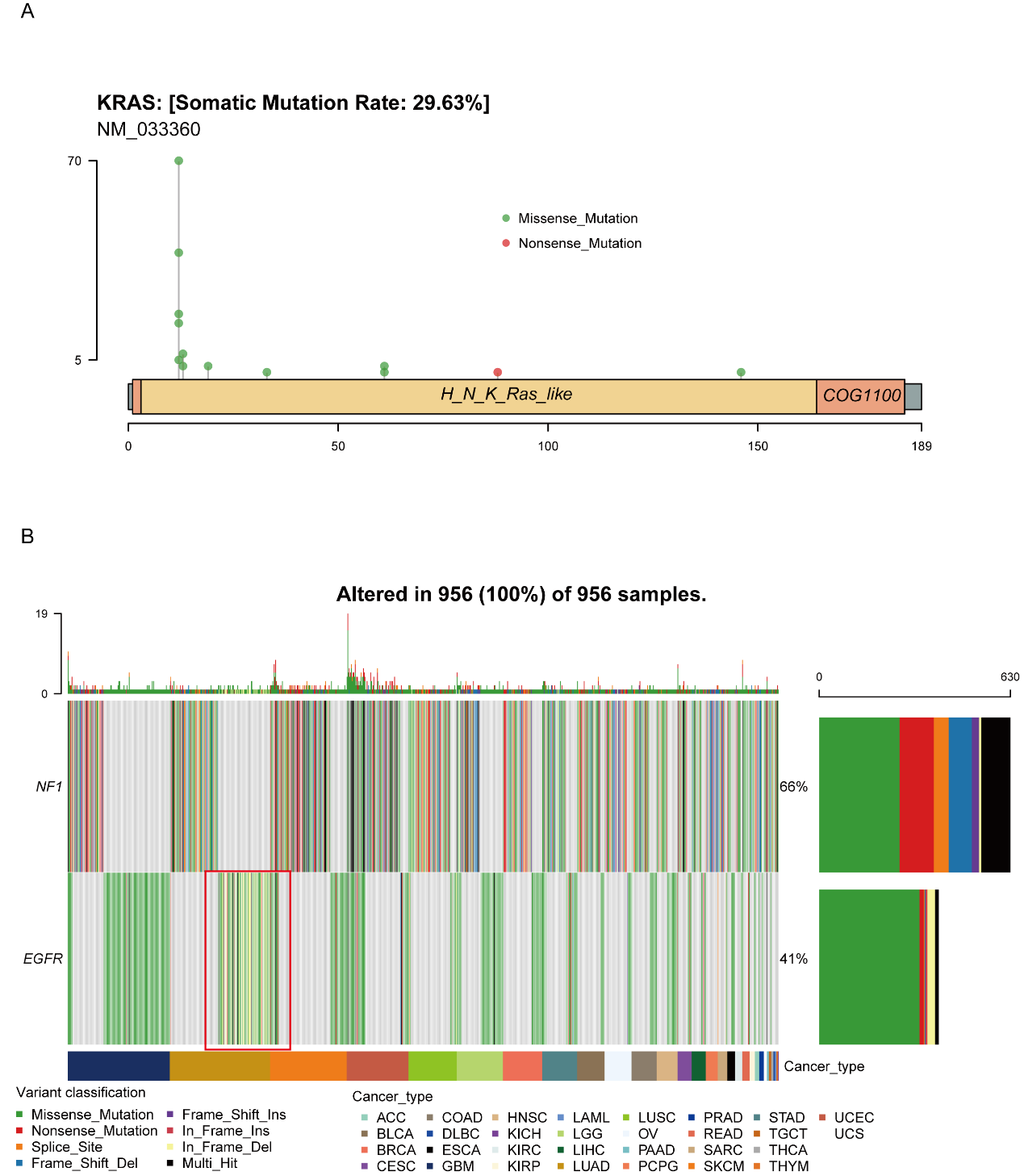


**Figure S2. SNV summary of RTK/RAS pathway. Supplement for Figure1.** (A) Lollipop plot showing the location and count of SNV on the coding region of *KRAS* in LUAD. (B) Distribution of SNV of *EGFR* and *NF1* in 33 cancer samples.


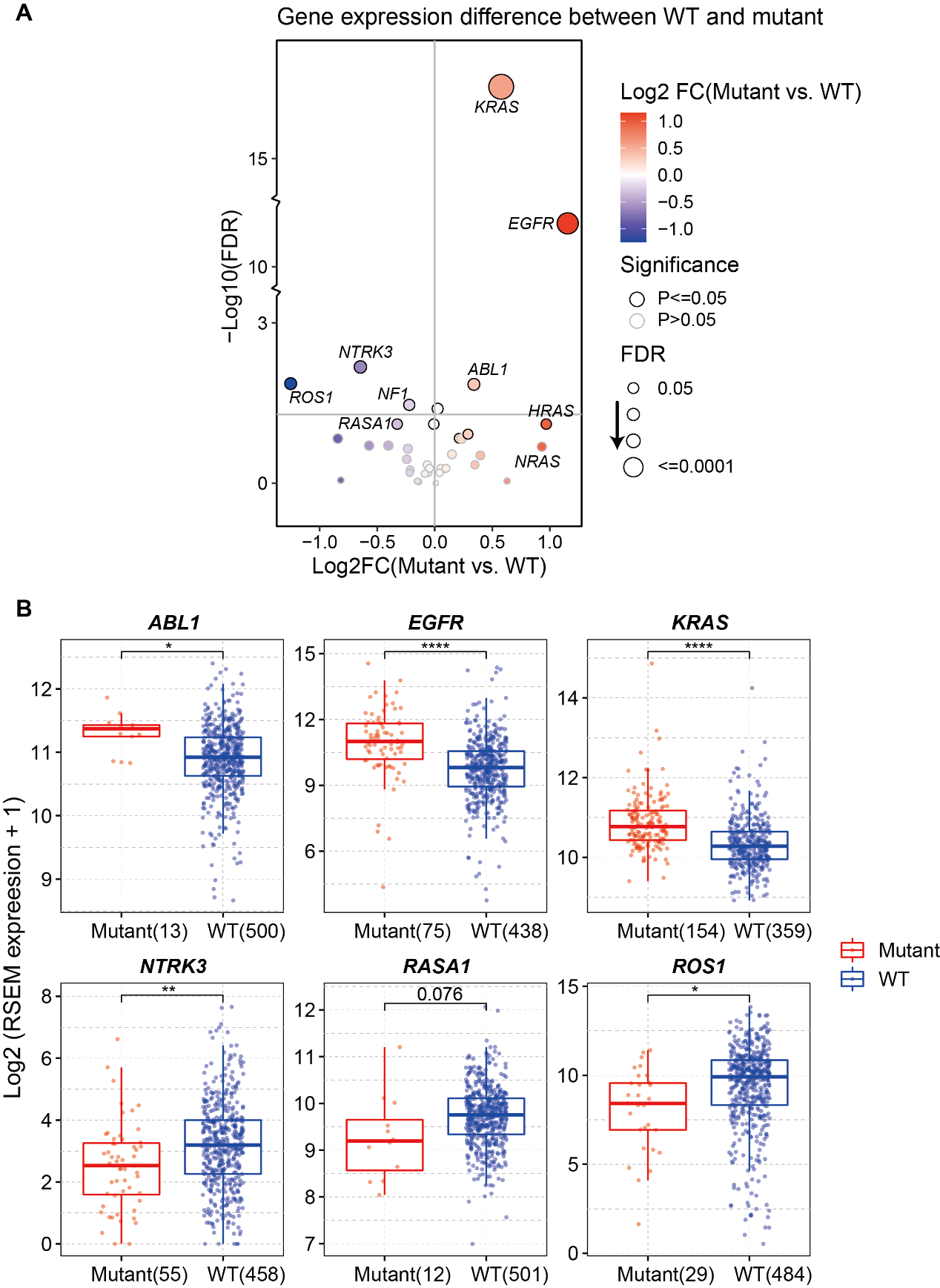


**Figure S3. The influence of RTK/RAS gene SNV on RTK/RAS mRNA expression.** (A) Summary of the differential expression of genes between WT and mutant. (B) Boxplot showing the comparison of mRNA expression between mutant and WT.


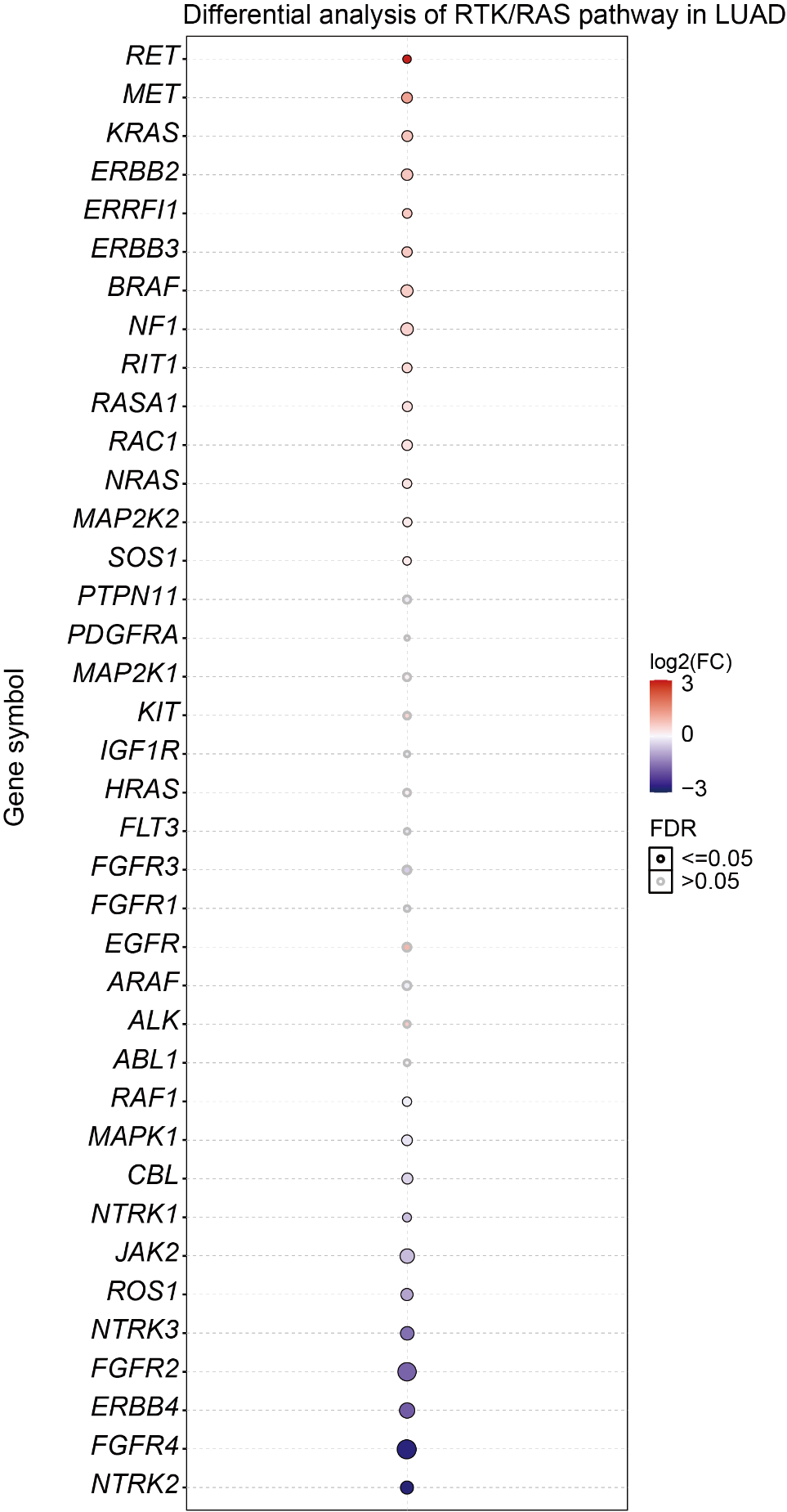


**Figure S4. Differential expression analysis for RTK/RAS pathway in LUAD.** The bubble plot shows the differentially expressed genes in LUAD. Blue and red bubbles represent downregulated and upregulated, respectively. Bubble size is positively correlated with the FDR significance. The black outline borders indicate FDR ≤ 0.05.

**
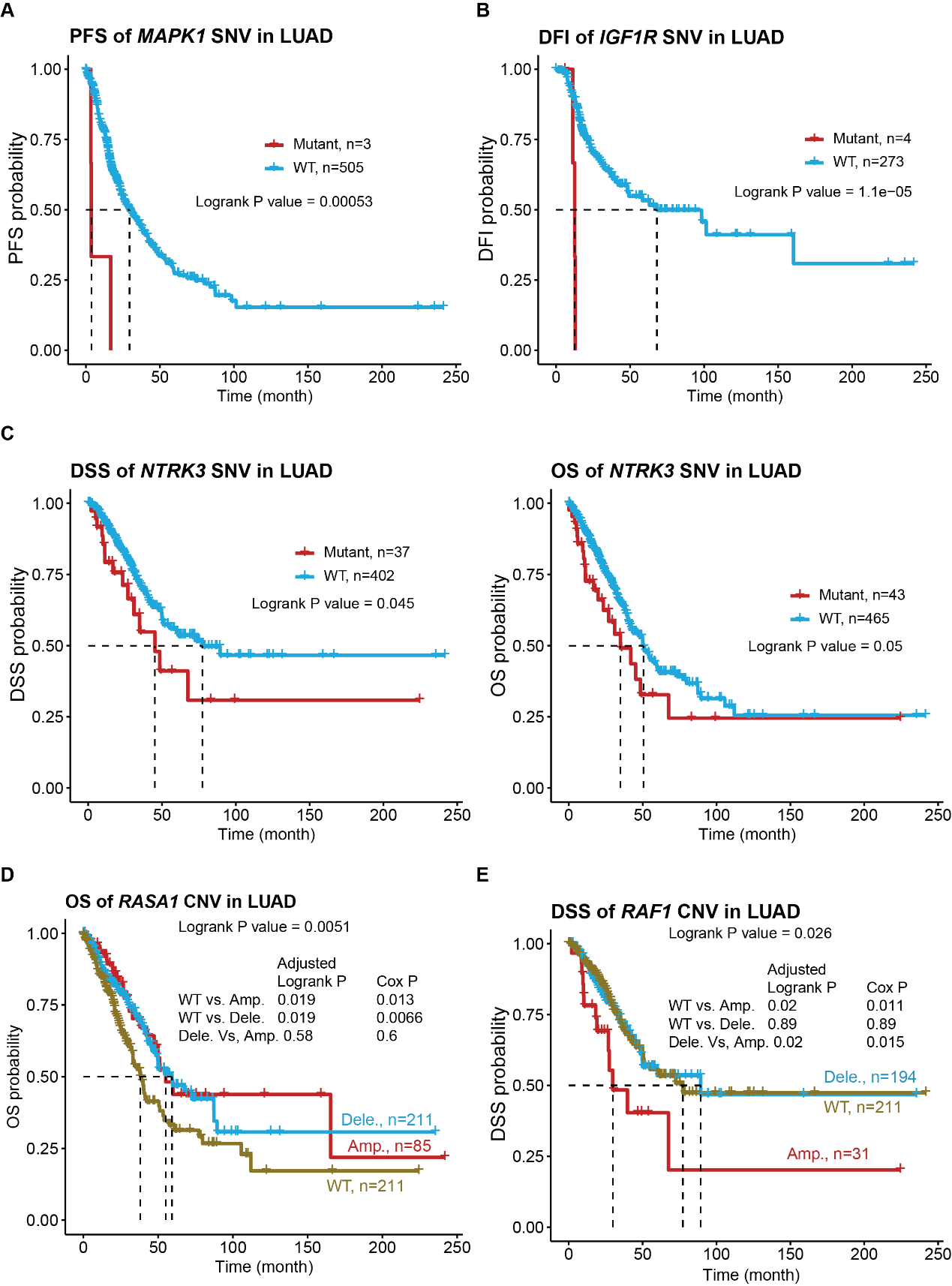
**

**Figure S5. K-M survival curves of genomic changes. Supplement for Figure 4.** (A) PFS difference between MAPK1 SNV and WT. (B) DFI difference between IGF1R SNV. (C) DSS and OS difference between NTRK3 SNV. (D) DSS difference between EGFR CNV. (E) DSS difference between RAF1 CNV.

**
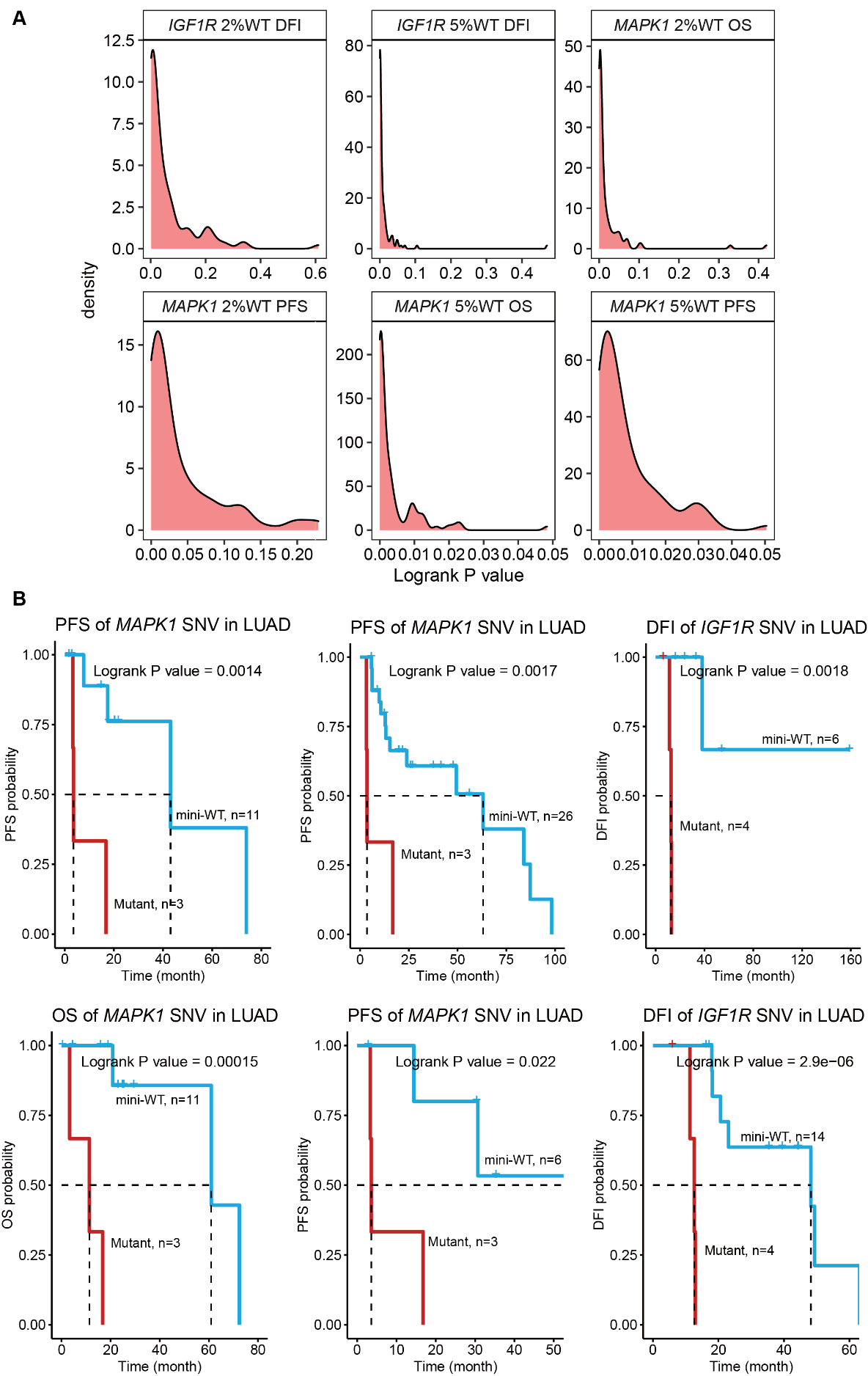
**

**Figure S6. Survival difference between** **“mini-WT” groups and *MAPK1*/*IGR1R* mutant groups.** We randomly selected 2% and 5% WT samples to construct“mini-WT” groups, each repeated 100 times. (A) The density distribution of Logrank P values in survival analyses, which compared “mini-WT” and MAPK1/IGR1R mutant group. (B) Survival difference between “mini-WT” and MAPK1/IGR1R mutant group. Several cases were shown, which were selected from the 100-times-repeated “mini-WT” selections.


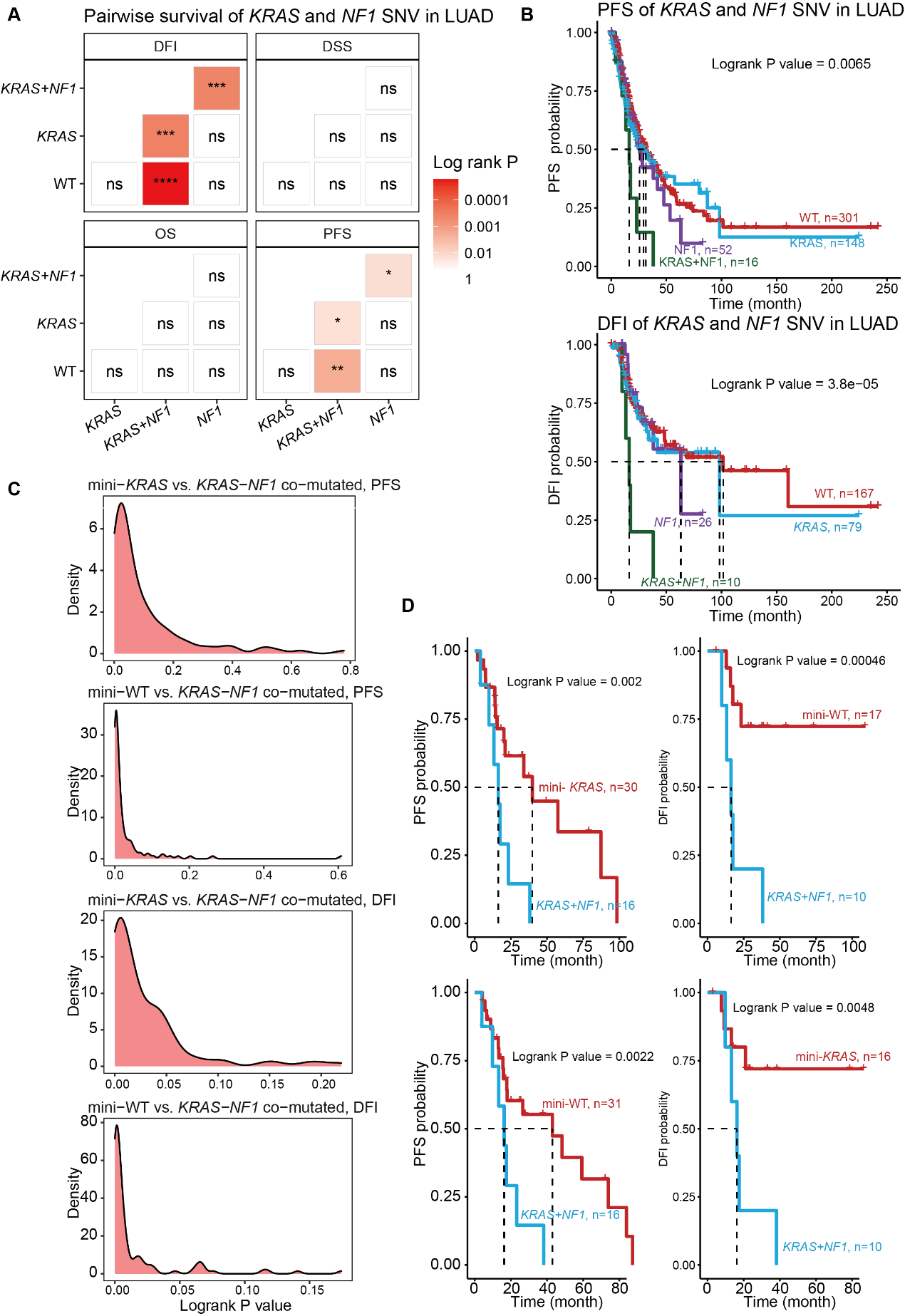


**Figure S7. Survival difference between *KRAS-NF1* co-mutated and others (WT or *KRAS* or *NF1* mutated).** (A) OS, PFS, DSS, and DFI survival difference between KRAS-NF1 co-mutated and others. The Log-rank P values were adjusted by FDR. (B) K-M survival curves between *KRAS* and *NF1* groups. (C) Survival difference between “mini-*KRAS*” or “mini-WT” groups and *KRAS-NF1* co-mutated group. We randomly selected part of samples to construct “mini” groups, each repeated 100 times. The density distributions of Log rank P values in these 100 iterations were presented. (D) Survival difference between “mini” groups and *KRAS-NF1* co-mutated group. Several cases that were selected from the 100 iterations were shown.


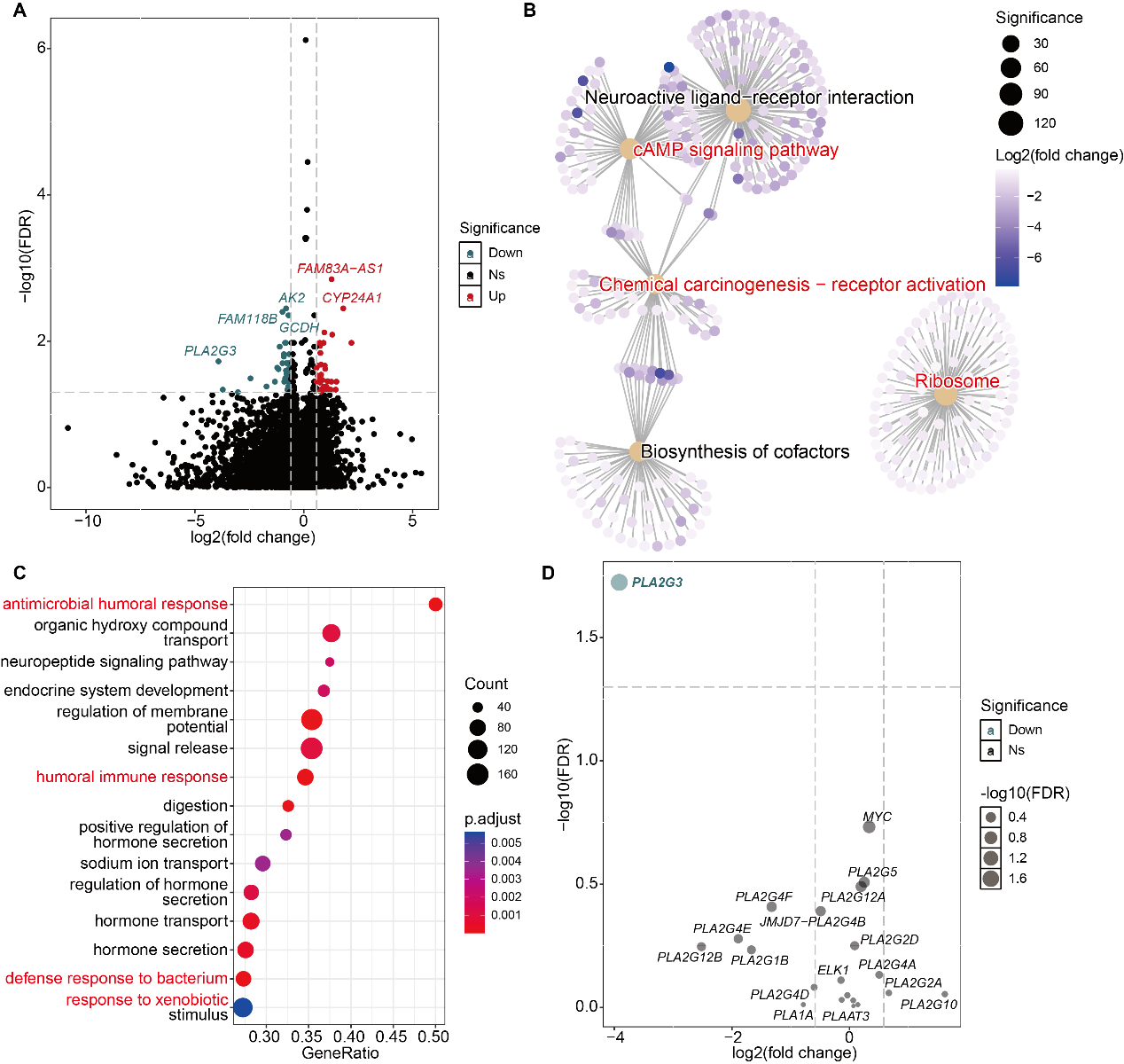


**Figure S8. Differential expressed genes between *KRAS*-*NF1* co-mutated LUAD samples and WT LUAD samples.** (A) Volcano plot of differential expressed genes. (B) KEGG gene set enrichment analysis for all genes ranked by fold change. (C) GO biological process gene set enrichment analysis for all genes ranked by fold change. (D) Differential expression summary of 22 RTK/RAS pathway downstream genes.


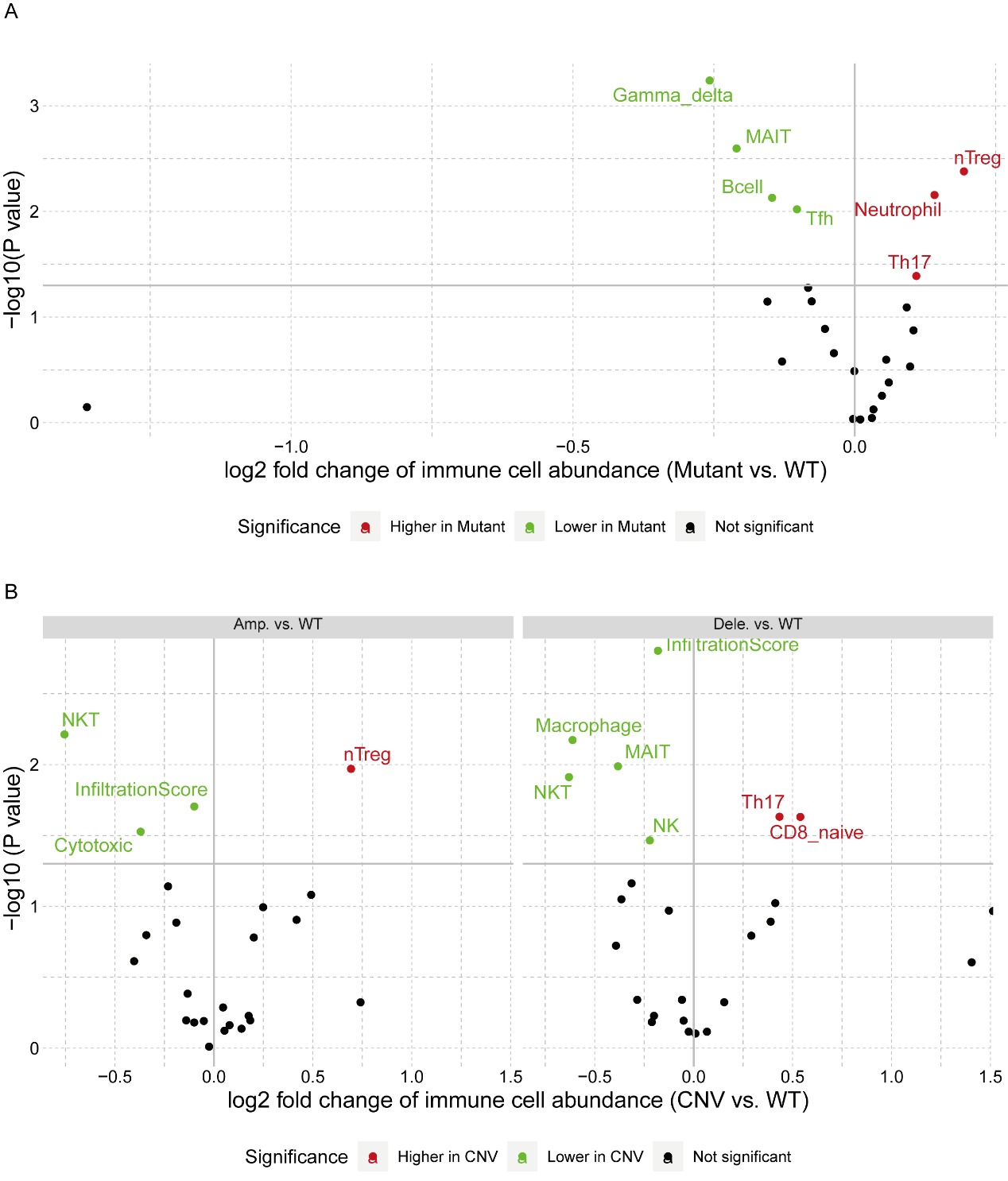


**Figure S9. The association of gene set SNV/CNV with immune cell infiltrations.** Difference of immune cell abundance between (A) SNV (mutant and WT) and (B) CNV (Amp.: amplification, Dele.: deletion, and WT) groups.


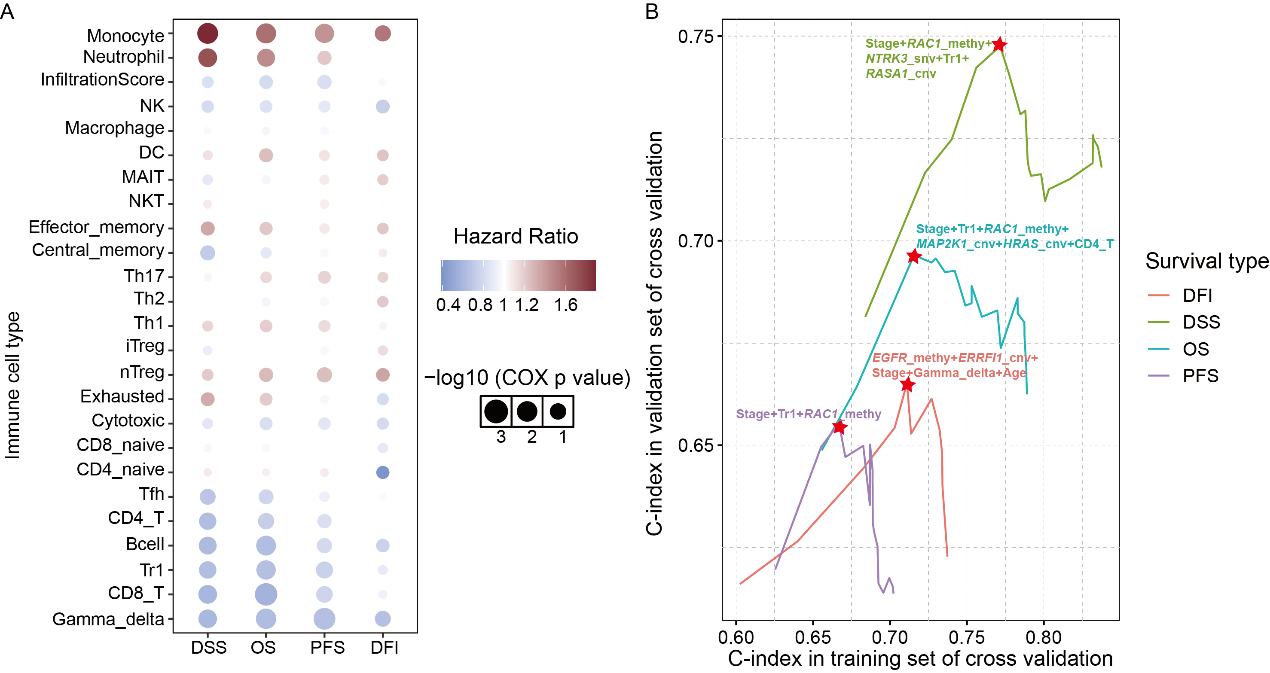


**Figure S10. The association of immune cell abundance with survival. Supplement for Figure 6.** (A) Survival analysis for the abundance of 24 immune cells. Blue and red bubbles represent hazard ratios (high abundance vs. lower abundance) <1 and >1, respectively. Bubble size is positively correlated with the FDR significance. Related to Table S7E. (B) The average C-index of each model in 10-fold cross-validation. The red star pointed out the performance of the final selected model in cross-validation. Related to Table S12.
